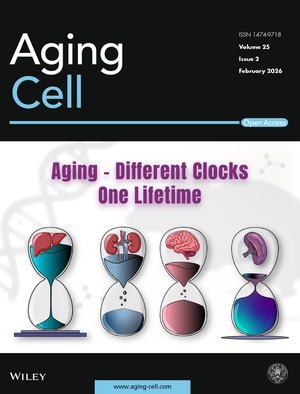# Additional Cover

**DOI:** 10.1111/acel.70401

**Published:** 2026-02-15

**Authors:** Sarah Morsy, Enzo Scifo, Kan Xie, Kristina Schaaf, Jenny Russ, Stefan Paulusch, Elena De Domenico, Paolo Salomoni, Daniele Bano, Dan Ehninger

## Abstract

The cover image is based on the article *Deciphering the Transcriptomic Signatures of Aging Across Organs in Mice* by Sarah Morsy et al., https://doi.org/10.1111/acel.70357.